# Lynch syndrome mutations in Poland and the Baltic States

**DOI:** 10.1186/1897-4287-13-S1-A11

**Published:** 2015-09-09

**Authors:** Dagmara Dymerska, Jan Lubinski, Grzegorz Kurzawski

**Affiliations:** 1 Department of Genetics and Pathology, International Hereditary Cancer Center, Pomeranian Medical University, Szczecin, Poland

## 

Lynch syndrome is a hereditary cancer syndrome that accounts for up to 15% of all colorectal cancers diagnosed at a young age. The disease is caused by germinal mutations in mismatch repair genes. To date, hundreds of small pathogenic and possibly pathogenic DNA variants (small insertions, deletions and substitutions) have been described for Lynch syndrome worldwide. Because of the variety of small changes in mismatch repair genes only full screening (exon by exon) by DHPLC/sequencing or HRM/sequencing seems to be the appropriate screening option. However, full screening is still too expensive and time consuming. A solution that uses the opportunity to reduce costs and improve diagnosis is the detection of recurrent mutations. This approach has been successfully applied as a pre-screening method. We have compared all identified changes in MMR genes detected to date from populations emanating from Estonia, Latvia, Lithuania and Poland to ascertain if there are any similarities in the mutation spectra that can be used in designing a rapid diagnostic test for recurrent mutations characteristic for the Eastern Baltic Sea region.

